# A Quantitative Analysis of Influencing Parameters for Cure Torque in Silica-Filled SSBR Compounds

**DOI:** 10.3390/polym18202596

**Published:** 2026-10-21

**Authors:** Anmol Aggarwal, Fabian Grunert, Sybill Ilisch, Anke Blume

**Affiliations:** 1 Elastomer Technology and Engineering, University of Twente, Drienerlolaan 5, 7522 NB Enschede, The Netherlands; a.aggarwal@utwente.nl (A.A.);; 2 Synthos Schkopau GmbH, Str. E 17, 06258 Schkopau, Germany

**Keywords:** cure torque, SSBR, silica, silane, filler flocculation, vulcanization

## Abstract

The maximum torque in the cure curve is commonly used as an indicator of crosslink density (CLD) in rubber compounds. However, in silica-filled systems, this relationship is affected by additional contributions to this torque, including filler–filler interactions, silane–mediated reactions (where silica-silane-polymer couplings contribute to the overall crosslink density), and interactions between silica and the accelerators. These contributions result in a higher complexity in the interpretation of cure curves in silica-filled compounds compared to carbon black-filled systems, while limiting the validity of maximum torque as a direct measure of crosslink density. This paper aims to subdivide the maximum torque of silica–filled styrene-butadiene rubber (SSBR) compounds into its constituent contributions arising from compound viscosity, filler–filler and filler–polymer interactions, and polymer crosslinking. Compounds based on a functionalized SSBR designed for chemical silica coupling were prepared at varying silica loadings, and the influence of each factor on the cure torque was investigated. At low silica loading, cure torque is governed primarily by intrinsic polymer characteristics, with a dominant contribution from polymer crosslinking. With increasing silica loading, the contribution of filler–filler and filler–polymer interactions increases significantly and progressively dominates the torque response, thereby reducing the apparent contribution of polymer crosslinking. Between 30 and 90 phr silica, while the estimated contribution of chemical crosslinking to the maximum torque decreased from about 37% to 28%, the contribution of filler–filler interactions increased from about 15% to 28%. Furthermore, without silane, the storage modulus at low strain was up to ten times higher than that of the corresponding compounds with silane, although the crosslink density remained close to that of the unfilled compound. Consequently, in highly filled silica compounds, cure torque cannot be directly correlated with crosslink density. These findings estimate the relative contributions of individual interactions to cure torque development in silica–filled rubber compounds, indicating that crosslink density alone cannot account for torque behavior at high filler loading.

## 1. Introduction

Vulcanization is essential for achieving the mechanical properties required of rubber products, and cure characteristics obtained by rheometer are commonly used to monitor this process. Specifically, the maximum torque in the cure curve is widely used as an indicator of crosslink density [1]. However, this relationship becomes more complex in silica-filled compounds. The hydrophilic surface of silica, in contrast to the hydrophobic nature of rubber, makes it challenging to disperse silica uniformly within the rubber matrix. This incompatibility promotes the formation of larger silica clusters during mixing and curing. To address this, coupling agents are used to reduce clustering and to chemically couple rubber chains to silica particles. These agents typically contain two functional groups: alkoxy groups, which react with silanol groups on the silica surface, and sulfide groups, which can form covalent bonds with the polymer. In high-vinyl solution styrene–butadiene rubber (SSBR), the sulfide groups preferentially react with vinyl double bonds present as side groups along the polymer backbone. In natural rubber (NR), however, such vinyl groups are absent, since the polymer backbone consists of isoprene units without side-chain unsaturation. The coupling in NR occurs preferentially at allylic positions along the main chain, where the C–H bonds adjacent to the backbone double bonds are more reactive toward radical attack [2,3,4].

Silica clustering, or flocculation, is a key phenomenon influencing the performance of silica-filled rubber compounds. In typical passenger car tire tread formulations, flocculation is detrimental as it reduces silica dispersion and its reinforcing effect. This occurs when polar hydroxyl groups on silica surfaces interact, particularly in the absence of coupling agents, forming clusters that reduce the available surface area for rubber–filler interactions. Consequently, the compound exhibits reduced reinforcement and inferior dynamic properties [5].

In unfilled compounds, the torque development during curing is primarily governed by the formation of chemical crosslinks between polymer chains. In silica-filled systems, however, additional interactions might influence this torque as well. The possible interactions are the strong filler–filler interactions (including flocculation) and polymer–filler coupling via silane. Additionally, the intrinsic viscosity of the compound also contributes to the measured torque, as viscosity is itself influenced by the degree of filler flocculation. Each of these factors might contribute to the torque response and may lead to misinterpretation of the extent of crosslinking [5,6]. Several studies have investigated the individual impact of these interactions on cure torque. Lin et al. [7] investigated silica flocculation during mixing and cure, and filler–rubber coupling, and found that silica flocculation was significantly reduced by silane addition, with the degree of suppression depending on the silane type and concentration. Similarly, Mihara et al. [5] observed a pronounced torque rise at the beginning of the vulcanization process in silica-filled compounds, which was associated with flocculation. They further showed that the flocculation rate decreases with an increasing amount of rubber chemically bound to the silica via the coupling agent, and increases with increasing silica surface area and temperature. Together, these parameters influence the MDR profile and complicate the distinction between mechanical reinforcement and chemical network formation [7,8]. This means that the relative contribution of filler–filler interactions, filler–polymer coupling, compound viscosity, and chemical crosslinking to the final cure torque remains unclear, especially in systems where these mechanisms occur simultaneously.

**Figure 1:**
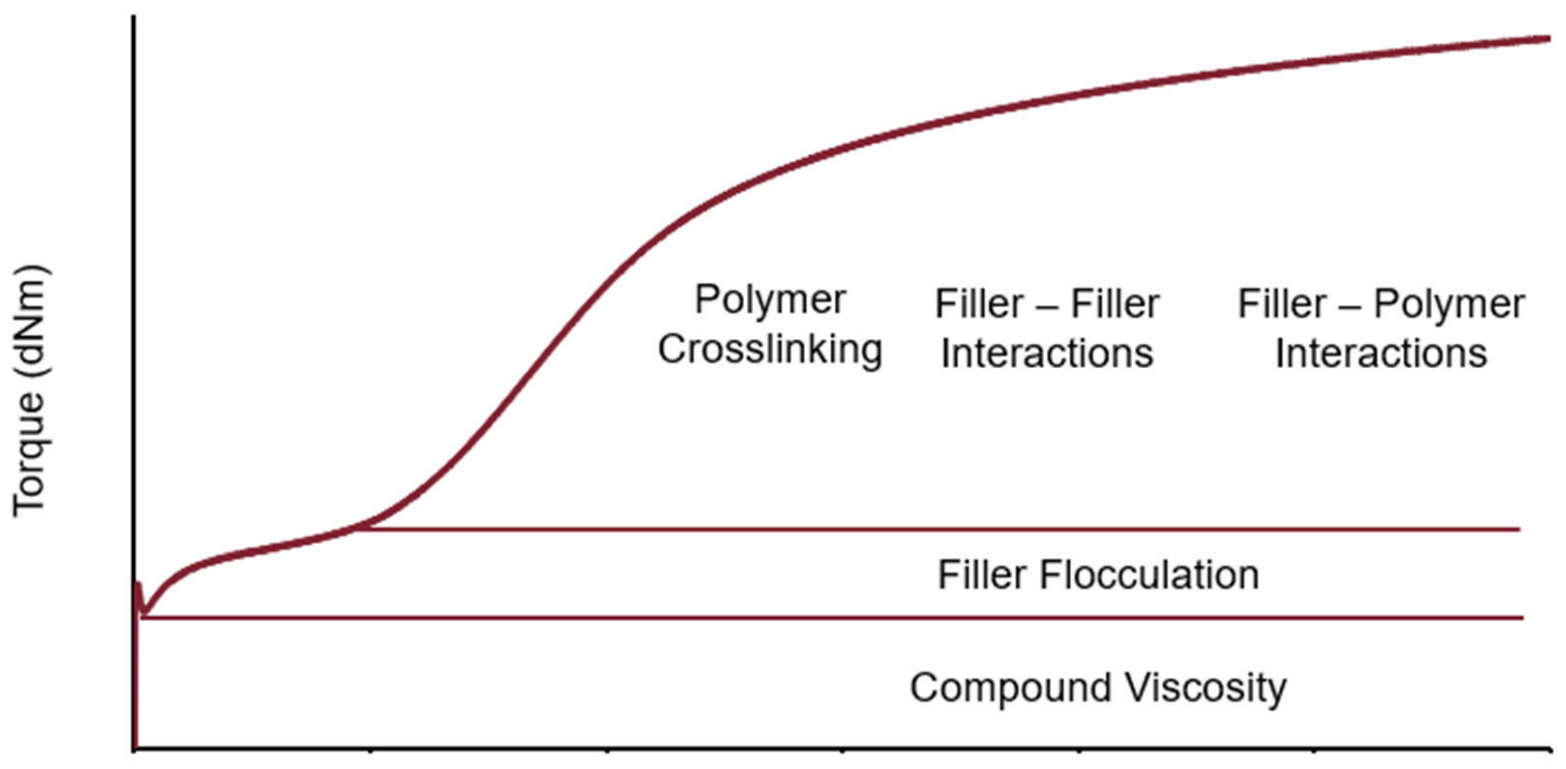
Contributions of possible interactions to the cure curve in a silica-filled compound.

This paper aims to subdivide the maximum cure torque of silica-filled SSBR compounds into its individual contributions and hence clarify the extent to which the commonly assumed torque–crosslink density correlation holds under these conditions. Figure 1 shows the schematic representation used in this study to break down the cure torque into its individual contributions, illustrated here for a silica-filled SSBR compound at high loading. The minimum torque, observed at the initial dip in the cure curve, is related to the compound viscosity, with higher viscosity leading to higher minimum torque [9]. This is particularly evident in functionalized polymers, where end-chain functionalization increases compound viscosity [10]. Filler flocculation contributes to the initial torque increase (“bump”), as described in the literature [5,8]. The contributions to the maximum torque arise from polymer crosslinking, silica–polymer coupling, and filler–filler interactions. Their relative importance depends on factors such as filler loading, dispersion quality, coupling agent efficiency, polymer viscosity and functionalization, mixing conditions, curing temperature, and curing system [9,11].

To determine these individual contributions, a structured experimental matrix was designed in which components such as silane coupling agents and silica were selectively excluded. The unfilled compound without silane represents the baseline polymer response, while silica-filled compounds allow for evaluation of filler-related interactions in the compounds. These formulation variations were combined with a set of complementary characterization techniques, including Mooney viscosity, cure torque measurements, swelling-based crosslink density, bound rubber content, and storage modulus at low strain (*G*′ at 0.56%) as a measure of filler–filler interactions, each targeting a specific mechanism contributing to cure torque. This is the first study that combines these independent measurements to estimate the individual contributions of compound viscosity, filler–filler interactions, filler–polymer coupling, and chemical crosslinking to the cure torque.

By comparing these formulations, this work provides insight into how different interactions influence the cure curve. This knowledge is essential for the accurate interpretation of cure torque results. This allows the design of rubber compounds for demanding applications where controlled curing behavior is critical, such as tire tread formulations. Furthermore, an improved understanding of the factors influencing cure torque supports material selection and formulation strategies, contributing to the overall development and optimization of rubber materials and high-performance tire applications.

## 2. Materials and Methods

### 2.1. Raw Materials and Formulation

For this investigation, SSBR SPRINTAN^®^ 4602 from Synthos Schkopau GmbH (Schkopau, Germany) with a Mooney viscosity ML (1 + 4) 100 °C of 65 MU was used as the base polymer. It is an end-chain functionalized rubber that can chemically react with silica and improve the mechanical and dynamic properties of the compounds [12]. Table 1 provides an overview of the ingredients used in this work.

To reinforce the compounds, precipitated silica (ULTRASIL^®^ 7000 GR from Evonik Industries AG, Essen, Germany), which is a highly dispersible grade of silica with a specific surface area (CTAB) of 160 m^2^/g was used. A coupling agent bis(triethoxysilylpropyl)disulfide TESPD (Si 266^®^ from Evonik Industries AG, Essen, Germany) was used to chemically link the non-polar polymers and the polar silica, thus enhancing the polymer–filler interaction. Other ingredients such as Treated Distillate Aromatic Extracted oil (Vivatec 500 from Hansen & Rosenthal KG, Hamburg, Germany), N-(1,3-Dimethylbutyl)-N′-phenyl-p-phenylenediamine (6PPD) (Vulkanox 4020/LG from Lanxess Deutschland GmbH, Cologne, Germany), paraffin wax (Protektor G 3108 from Paramelt B.V., Heerhugowaard, The Netherlands), carbon black (N330 from Apollo Tyres B.V., Enschede, The Netherlands), vulcanization system comprising the activators stearic acid (Edenor ST1 GS from Emery Oleochemicals GmbH, Düsseldorf, Germany) and zinc oxide (ZnO RS RAL 844 C from Arnsperger Chemikalien GmbH, Cologne, Germany), α-Sulphur from Eastman Chemical Corporation, Langenfeld, Germany, N-tert-butyl-2-benzothiazolesulfenamide (TBBS) (Rhenogran TBBS-80 from Lanxess Deutschland GmbH, Cologne, Germany) as the primary accelerator, and N,N′-Diphenylguanidine (DPG) (Rhenogran DPG from Lanxess Deutschland GmbH, Cologne, Germany) and tetrabenzyl thiuram disulfide (Richon TBZTD OP from Dalian Richon Chem Co., Ltd., Dalian, China) as secondary accelerators were used. The silica loadings of 30, 60 and 90 phr were chosen below and above the percolation threshold of approximately 45 phr, which was determined in previous work for compounds based on the same SSBR [13]. In that work, two consecutive strain sweeps were performed on uncured compounds at different silica loadings. While below the percolation threshold, both sweeps overlap, above the percolation threshold, the storage moduli of the two sweeps differ, therefore indicating the formation of a continuous filler network. As seen in Table 2, the silane amount was adjusted according to Equation (1) [14]:

(1) *TESPD* (*phr*) = 4.7 *×* 10^−4^ *×* (*CTAB*)*_silica_*
*×* (*phr*)*_silica_*

Similarly, the amount of TDAE was adjusted according to Equation (2):

(2) *TDAE* (*phr*) = 0.3125 *×* (*phr*)*_silica_*

A total of seven compounds were prepared and analyzed, consisting of one unfilled compound and six silica-filled compounds, as shown in Table 2. The filled systems were divided into two sets: compounds without silane and compounds containing all ingredients (‘All in’). For the compounds without silane, three compounds containing 30, 60, and 90 phr silica were prepared, with oil content adjusted to maintain comparable compound consistency. The ‘All in’ ones included three compounds at the same silica loadings (30, 60, and 90 phr), with both silane and oil contents adjusted accordingly. The oil content was increased proportionally with silica loading to maintain processable compound viscosities. Therefore, the effects of changes in silica loading and oil content cannot be fully separated within one compound series. However, the compounds without silane and the ‘All in’ compounds contain the same amounts of silica and oil at each loading, and hence, comparisons between the two series at equal loading are not affected by the oil content. This experimental design enables a systematic evaluation of filler-related interactions and their contribution to cure torque.

### 2.2. Mixing Process

The ingredients were mixed in a three-stage mixing process in a 390 mL Brabender Plasticorder 350 S internal mixer (Brabender GmbH & Co. KG, Duisburg, Germany) with tangential rotors (N-rotor geometry), followed by sheeting on a two-roll mill (Polymix 80T, Servitec Maschinenservice GmbH, Wustermark, Germany). The rubber sheets were stored for 24 h before the subsequent mixing stages, and the fill factor was reduced by 3 percentage points after each stage to account for material loss. The additional second mixing stage, contrary to the typical industrial practice of using a two-stage mixing process, is to improve the extent of the silanization reaction and ensure an effective mixing process. DPG was added as an accelerator in the second stage to additionally shield the silica surface and reduce the potential adsorption of the main accelerators TBBS and TBzTD in the final mixing stage. Table 3 shows the mixing procedure and the mixing conditions.

### 2.3. Cure Characteristics

The cure characteristics of the compounds were measured at 0.5° (or 7.2%) strain and a frequency of 1.67 Hz at 160 °C for 30 min in the Rubber Process Analyzer (RPA) from TA Instruments (New Castle, DE, USA) according to ASTM D5289-95 [15]. The measurement was used to determine the minimum and maximum torque (*S*′*_min_*, *S*′*_max_*) and to assess the progression of the vulcanization reaction. The cure characteristics were determined in a single measurement per compound. For the crosslink density measurements, the compounds were vulcanized in a hydraulic press (Wickert WLP 1600, Landau, Germany) at 160 °C and 100 bar for 30 min. The same vulcanization time was applied to all compounds instead of the individual t_90_ to overcome the limitations from the marching modulus in the cure curves of the silica-filled compounds.

### 2.4. Characterization

#### 2.4.1. Mooney Viscosity

The Mooney viscosity of the rubber samples was measured in the Mooney Viscometer 2000 from Alpha Technologies (Hudson, OH, USA). The measurement was performed by determining the Mooney ML (1 + 4) viscosity at a temperature of 100 °C according to the ISO 289 standard. ML stands for “Mooney large rotor,” while (1 + 4) refers to the test procedure: 1 min of preheating followed by 4 min of measurement at a constant rotor speed of 2 revolutions per minute (rpm) [16]. The Mooney viscosity was determined in a single measurement per compound.

#### 2.4.2. Crosslink Density

The crosslink density (CLD) of the vulcanized rubber compounds was determined using the equilibrium swelling method, which provides an indirect measure of the number of crosslinks per unit volume of the rubber. This is based on the principle that more highly crosslinked rubber networks swell less in a solvent due to restricted molecular mobility. Approximately 1.5 g of each sample was extracted in a Soxhlet extraction unit with acetone for 24 h, followed by drying at 60 °C in a vacuum oven for 12 h. The extraction removes low-molecular, non-rubber-soluble substances, such as the zinc salt of the accelerator and its decomposition products, zinc fatty acid soaps, antioxidants, polymerization aids, and oils. The dried compound was cut into five specimens per sample (∼50 mg each). The cut samples were put in vials, filled with toluene, and left to swell for 7 days. The final swollen weight was measured and used to calculate the swelling ratio, an intermediate step for determining CLD by Equation (3) [17]:

(3) $$\mathrm{Swelling}\;\mathrm{Ratio}\;(\%)=\frac{m_{2}-m_{1}}{m_{1}}\times 100$$

where *m*_1_ and *m*_2_ were the masses of the vulcanized sample before and after swelling, respectively.

The swelling ratio was then used to calculate the crosslink density (ν) using the modified Flory–Rehner Equation (Equation (4)), which accounts for the thermodynamic interaction between the polymer and solvent, as well as the elasticity of the crosslinked network, and provides crosslink density with filler correction:

(4) $$\nu =-\frac{\ln(1-V_{r})+V_{r}\;+\chi V_{r}^{2}}{V_{0}(V_{r}^{\frac{1}{3}}-V_{r}/2)}$$

In this equation, ν represents the crosslink density in mol/m^3^, while *V_r_* is the volume fraction of rubber in the swollen gel. The parameter *χ* denotes the polymer–solvent interaction coefficient, and *V*_0_ is the molar volume of the solvent, which for toluene is typically taken as 106.9 cm^3^/mol. *V_r_* was calculated for each specimen as follows: The mass of the non-swelling fillers (silica, zinc oxide and carbon black) was calculated from the formulation, taking into account the mass loss during acetone extraction, and subtracted from the dried specimen mass to obtain the rubber mass “*m_r_*”. The equilibrium mass swelling *Q_m_* = (*m_s_* − *m_d_*)/*m_r_*, with m_s_ and m_d_ as the swollen and dried specimen masses, was converted into the volume swelling *Q_v_* = *Q_m_*·ρ_r_/ρ_s_, with ρ_r_ = 0.94 g/cm^3^ and ρ_s_ = 0.862 g/cm^3^ as the densities of the rubber and toluene. *V_r_* was then obtained as *V_r_* = 1/(1 + *Q_v_*). For this study, *χ* was assumed to be 0.378, which is appropriate for SBR–toluene systems [18].

#### 2.4.3. Bound Rubber

When fillers are mixed with rubbers, a portion of the rubber chains is bonded to the active sites on the filler surface. These are high-energy sites, such as silanol groups and siloxane chains in silica, which can be coupled chemically. If the uncured rubber is extracted from the filled uncured rubber compound with a suitable solvent (such as toluene or cyclohexane), the rubber molecules bound on this surface remain in the form of a rubber-filler gel. The bound rubber in the gel depends on the type of rubber, the structure and specific surface area of the filler, the mixing procedure, as well as the type of solvent used. The total bound rubber content can be calculated by Equation (5):

(5) $$\mathrm{Total}\;\mathrm{Bound}\;\mathrm{Rubber}\;\mathrm{Content}\;(\%)=\frac{W_{\mathrm{fg}}-W\left(\frac{m_{f}}{m_{f}+m_{p}}\right)}{W\left(\frac{m_{p}}{m_{f}+m_{p}}\right)}\times 100$$

*W_fg_* is the weight of filler plus gel, *W* is the original weight of the specimen, and *m_f_* and *m_p_* are the weights of filler and polymer in the compound, respectively. For this work, approximately 0.2 g of the uncured rubber compound was cut into small pieces and immersed in toluene at room temperature for 5 days, while the toluene was renewed every day. Thereafter, the samples were removed from the toluene, dried at 80 °C for 24 h and weighed. To distinguish physically adsorbed from chemically bound rubber, the measurement was repeated under ammonia atmosphere, which disrupts physical interactions such as hydrogen bonds between rubber and silica. The rubber remaining after this treatment is referred to as chemically bound rubber, and the physically bound rubber content was calculated as the difference between the total and the chemically bound rubber content [19,20].

#### 2.4.4. Filler–Filler Interaction (Storage Modulus at Low Strain)

The extent of filler–filler interaction in the uncured compounds was evaluated by using the RPA mentioned in Section 2.3, at 100 °C, with a strain of 0.56% and a test time of 14 min, including a 2-min pre-heating period. The measurement temperature was selected according to a typical industrially employed extrusion temperature [21]. The storage shear moduli were recorded at different measurement times. According to Mihara et al. [5], the flocculation of silica clusters is observed by the change in storage modulus (*G*′) at low strain under isothermal conditions.

Instead of calculating the rate of this change, the storage modulus, *G*′, recorded at the end of the 12-min isothermal measurement period was used as an indicator of the extent of filler–filler interaction. At low strain, *G*′ is strongly influenced by the reformed filler network structure that develops during this isothermal period following the initial breakdown of the filler network during pre-heating [5]. By the end of the 12-min measurement, since the filler network reforms, the *G*′ value at this point indicates the strength of the reformed filler network. A higher *G*′ value indicates stronger filler–filler interactions, due to silica clustering and the formation of a more extensive filler network, which restricts molecular mobility and increases stiffness. The storage modulus *G*′ (0.56%) was determined in a single measurement per compound.

## 3. Results and Discussion

**Figure 2:**
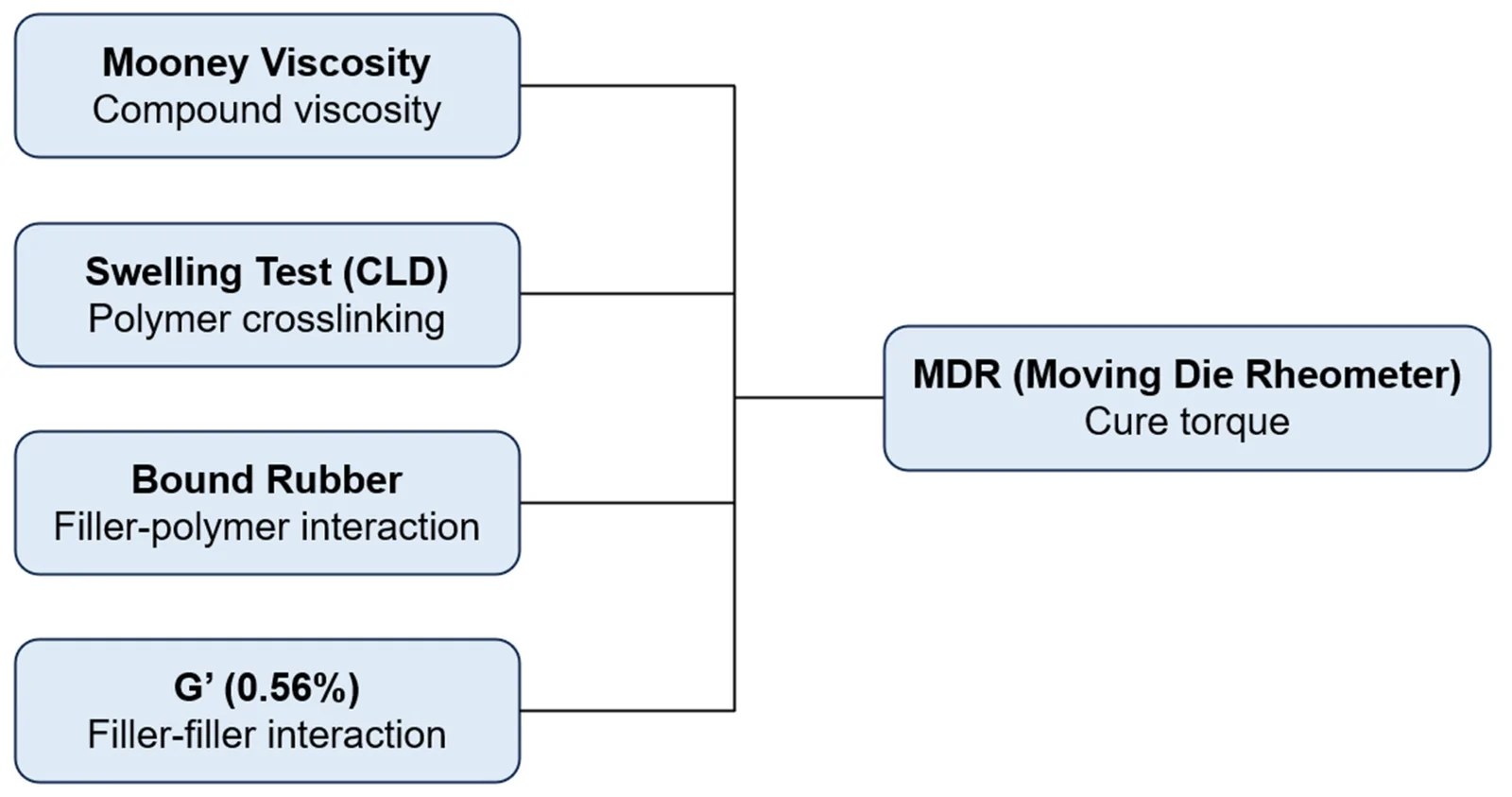
Flowchart of the measurements used to characterize the different contributions to the cure torque in silica-filled SSBR compounds.

To investigate the extent to which cure torque can be used as a measure of crosslink density in silica-filled SSBR compounds, the individual contributions of compound viscosity, filler–filler and filler–polymer interactions, and chemical crosslinking to cure torque were characterized. Since cure torque development results from the combination of these physical and chemical interactions, a single measurement method is insufficient to isolate their individual effects. Figure 2 shows a flowchart of the approach used to subdivide the cure torque into its individual contributions. Four measurement techniques were used: Mooney viscosity, swelling-based crosslink density, bound rubber content, and storage modulus at low strain (*G*′ at 0.56%), each targeting a specific mechanism contributing to cure torque.

The unfilled compound provides an indication of the chemical crosslink density without the influence of filler-related interactions. Filled compounds without silane help to understand the extent of filler–filler interactions in the absence of filler–polymer coupling reactions. Compounds containing all ingredients (‘All in’) reflect the combined contribution of all mechanisms. Comparing these ‘All in’ compounds for increasing silica loading further indicates the extent of filler–filler and filler–polymer interactions with increasing filler content.

### 3.1. MDR Curves

**Figure 3:**
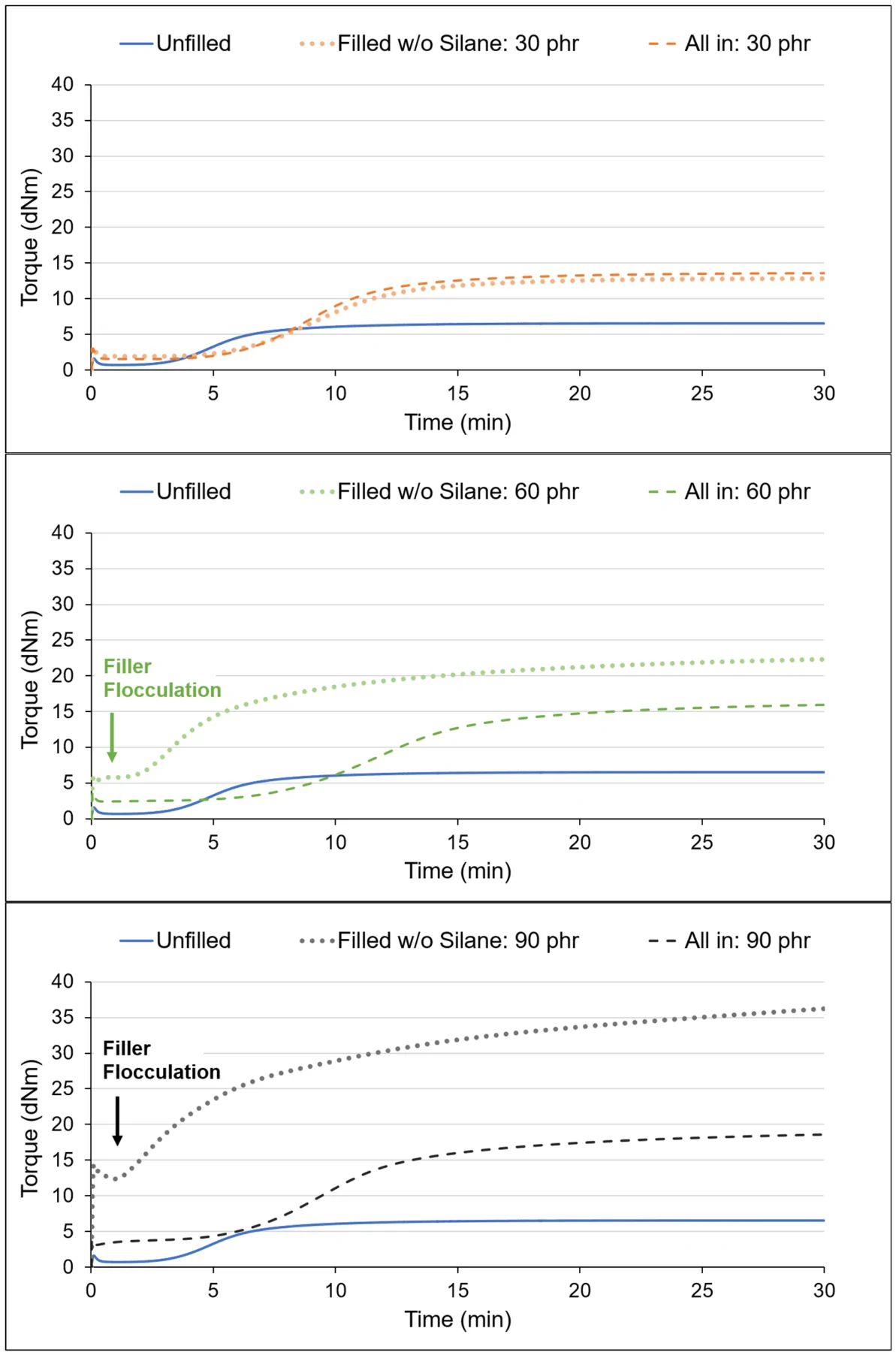
MDR cure curves of the unfilled compound together with the ‘Ffilled w/o Silane’ and ‘All in’ compounds at 30, 60, and 90 phr silica loading (top, middle, and bottom, respectively). All plots are shown on the same torque scale to allow a direct comparison between the silica loadings.

This section evaluates the vulcanization behavior of the compounds using MDR measurements done at 160 °C. MDR provides dynamic insight into the curing process, including the torque buildup due to crosslink formation, filler interactions, and the effect of compound viscosity on vulcanization. Figure 3 shows the MDR cure curves of the unfilled compound together with the ‘Filled w/o Silane’ and ‘All in’ compounds at 30, 60, and 90 phr silica loading (top, middle, and bottom, respectively).

For the unfilled compound, in the absence of silica and silane, the torque response reflects only the contribution of the polymer matrix and excludes filler-related effects. Therefore, the measured cure behavior primarily represents the influence of intrinsic compound viscosity and polymer crosslinking on torque development. The unfilled compound exhibits a typical vulcanization profile, characterized by an initial decrease in torque followed by a gradual increase toward a plateau region. The initial decrease in torque is associated with thermal softening of the compound at the curing temperature, while the subsequent increase results from the formation of the crosslinked polymer network during vulcanization. Compared to the filled compounds discussed later, the unfilled system shows relatively low torque values due to the absence of additional contributions from filler–filler and polymer–filler interactions. Since no reinforcing filler is present, polymer chain mobility remains relatively unrestricted, resulting in lower compound stiffness and lower torque values. Thus, the unfilled compound serves as a reference system for evaluating the influence of filler addition and filler-related interactions on the cure behavior of silica-filled compounds [22,23].

For both compound series (‘Filled w/o Silane’ and ‘All in’), the torque increases with increasing silica loading, indicating the increasing contribution of filler-related interactions to the cure behavior. This effect is particularly pronounced for the compounds without silane, where both the initial and final torque values increase substantially with silica loading. In the absence of silane, filler–filler interactions dominate, promoting flocculation and increasing compound stiffness. Consequently, the compounds without silane exhibit consistently higher torque values than the corresponding ‘All in’ compounds. The differences between the two systems become more evident above the silica percolation threshold (~45 phr) [13].

At 30 phr silica loading (Figure 3, top), below the percolation threshold, the torque curves of both systems remain relatively close to each other and to the unfilled compound, indicating limited filler–filler interactions at this loading, even in the absence of silane. However, at 60 phr silica loading (Figure 3, middle), differences between the two compound series are visible. The filled w/o silane compound shows a pronounced initial ‘bump’ in torque (green arrow) at the start of the cure curve, associated with silica flocculation [5], which is more pronounced than in the ‘All in’ compound. This indicates that above the percolation threshold, filler–filler interactions become increasingly dominant in the absence of silane. In contrast, silane suppresses the early torque increase caused by filler flocculation by promoting silica–polymer interactions.

At 90 phr silica loading (Figure 3, bottom), the torque response changes significantly. As observed at 60 phr, the filled w/o silane compound again shows a pronounced initial bump in torque associated with silica flocculation, and this bump is even more pronounced at 90 phr, particularly in comparison to the ‘All in’ compound at the same loading. Furthermore, the compound without silane shows markedly higher torque values and a continuous increase in torque throughout the measurement, indicating increasingly strong filler–filler interactions. These observations correspond well with Mihara et al. [5], who attributed an early torque rise in the cure curves of silica-filled compounds to silica flocculation. They further showed that the flocculation rate decreases with an increasing amount of rubber chemically bound to the silica via the coupling agent. The same trend is observed here: the bump is clearly present in the compounds without silane at 60 and 90 phr, but strongly reduced in the corresponding ‘All in’ compounds.

A characteristic feature observed in the cure curves is the delayed onset of torque increase, particularly for the ‘All in’ compounds as the filler loading increases. This behavior is typical for silica-filled systems and is commonly attributed to the adsorption of accelerators such as TBBS and DPG on the polar silica surface. The temporary reduction in accelerator availability delays the formation of active sulfurating species, thereby shifting the onset of crosslinking to later times. As curing progresses, desorption of the accelerators contributes to the gradual increase in torque. The delayed and gradual torque development contributes to the marching modulus behavior observed in the highly filled compounds, particularly at 90 phr silica. The continuous increase in torque without reaching a clear plateau indicates that, besides chemical crosslinking, additional physical processes continue during curing. In silica-filled systems, this behavior is associated with incomplete silica–rubber coupling, limited chain relaxation due to high filler rigidity, and ongoing rearrangement of silica clusters under cyclic deformation. Furthermore, poor silica dispersion can intensify this effect, as clustered silica domains promote stronger filler–filler interactions and continuous microstructural rearrangements during MDR measurements, resulting in a persistent increase in torque [24,25,26].

In contrast, the ‘All in’ compounds show lower torque values and a more gradual increase in torque with increasing silica loading. The presence of silane reduces direct silica–silica interactions by promoting silica–polymer interactions, thereby reducing the compound stiffness. Together with the lower *G*′ (0.56%) and Mooney viscosity, this indicates an improved filler micro-dispersion, although the dispersion was not characterized directly [27]. As a result, the torque increase becomes less pronounced and the cure curves approach a more stable plateau compared to the compounds without silane. Nevertheless, even at 90 phr silica, the ‘All in’ compound still exhibits relatively high torque values. This indicates that filler-related contributions remain significant despite the presence of silane. Since SPRINTAN^®^ 4602 is only end-chain functionalized, the number of functional groups remains limited relative to both the polymer molecular weight and the available silanol groups on the silica surface. Consequently, filler–filler interactions cannot be completely suppressed at high filler loadings. This is consistent with Lin et al. [7], who found that silane significantly reduces, but does not fully eliminate silica flocculation.

MDR results show that cure torque in silica-filled SSBR compounds is governed not only by chemical crosslinking but also increasingly by filler-related contributions as silica loading increases. Filler–filler interactions are dominant in the absence of silane, particularly above the percolation threshold, while silane suppresses these interactions through improved filler–polymer coupling reactions. These findings confirm that cure torque alone cannot be used as a direct measure of crosslink density in silica-filled systems, since contributions from filler–filler and filler–polymer interactions can become increasingly significant with at higher filler loadings.

### 3.2. Mooney Viscosity

Following the approach outlined in Figure 2, Mooney viscosity was used to determine the contribution of compound viscosity to cure torque, corresponding to the minimum torque in the cure curve. As an indicator of processability, Mooney viscosity describes the resistance of the uncured compound to flow and provides insight into the influence of formulation variables on compound stiffness prior to vulcanization. In silica-filled systems, polymer architecture, silane content, and filler loading strongly affect the viscosity and consequently influence the rheological response during curing, since the minimum torque in the cure curve is related to the compound’s starting viscosity [9]. Table 4 presents the Mooney viscosity results for the compounds. The ‘Filled w/o Silane’ compounds include silica but exclude the coupling agent, thereby highlighting the effects of filler addition without chemical bonding with the rubber. The ‘All in’ compounds contain all ingredients, representing fully formulated systems.

The viscosity of the unfilled compound is lower than that of the intrinsic polymer (65 MU). This reduction can be attributed to the presence of low-molecular-weight ingredients, such as stearic acid, which act as plasticizers, as well as possible polymer chain scission during mixing, which may reduce the effective molecular weight. In addition, the absence of filler reinforcement in the unfilled compound allows unrestricted polymer mobility, further contributing to the comparatively low viscosity.

The silica-filled compounds without silane (‘Filled w/o Silane’) exhibit significantly increased Mooney viscosity due to strong filler–filler interactions in the absence of coupling agents. These interactions promote the formation of silica clusters, increasing compound stiffness and restricting polymer mobility. At 30 phr, the filler loading remains below the percolation threshold (~45 phr) [13], preventing the formation of an extensive filler–filler network. At higher filler loadings (60 and 90 phr), this effect becomes more pronounced, resulting in significantly increased compound stiffness and correspondingly higher viscosity [3,28].

The ‘All in’ compounds show a more controlled and gradual increase in Mooney viscosity with increasing silica content. This reflects the role of the silane coupling agent in reducing filler–filler interactions, possibly improving filler micro-dispersion, and moderating compound stiffness. The effect becomes increasingly evident with increasing silica loading, where the presence of silane limits direct silica–silica interactions and promotes interactions between silica and the polymer. As a result, the increase in viscosity with filler loading is less pronounced compared to the compounds without silane. However, with increasing filler loadings, the compounds still exhibit high viscosities as compared to the unfilled compound, indicating strong silica–silica interactions and limited polymer mobility. Although SPRINTAN^®^ 4602 is end-chain functionalized to promote interaction with silica, the number of functional groups is low relative to both the polymer molecular weight and the available silanol (Si–OH) groups on the silica surface. As a result, polymer–filler interactions are not sufficient to fully suppress silica–silica interactions, especially at the highest filler loading. Consequently, the viscosity at high filler loadings is predominantly governed by these interactions, leading to a pronounced increase in compound stiffness. These trends are consistent with Fröhlich et al. [29] and Luginsland et al. [28], who showed that silanization hydrophobizes the silica surface and weakens the filler network, and with Lin et al. [7], who found that the addition of bis(triethoxysilylpropyl)tetrasulfide TESPT reduced the Mooney viscosity ML(1 + 4) at 130 °C of a compound with 80 phr silica from 192 to 90 MU. The comparatively small viscosity increase in the ‘All in’ compounds between 60 and 90 phr (88 to 92 MU) can additionally be attributed to the end-chain functionalization of SPRINTAN^®^ 4602, which Thiele et al. [10] reported to improve the filler dispersion.

**Figure 4:**
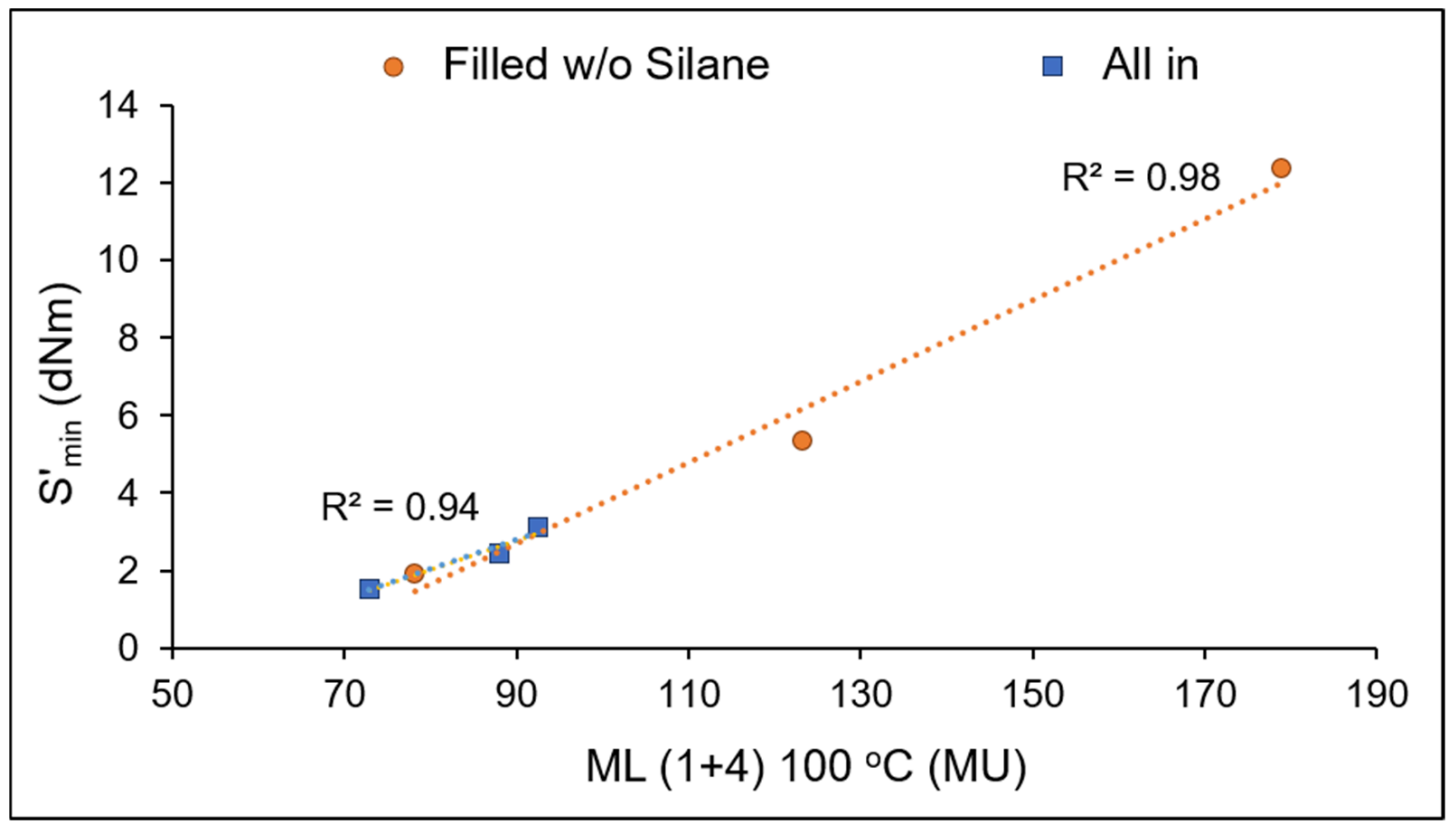
Correlation between the minimum torque (*S*′*_min_*) from MDR measurements and the Mooney viscosity of the ‘Filled w/o Silane’ and ‘All in’ compounds.

Figure 4 shows the correlation between the minimum torque (*S*′*_min_*) from MDR measurements and the Mooney viscosity of the ‘Filled w/o Silane’ and ‘All in’ compounds.

A positive correlation is observed for both compound series, with R^2^ values of 0.94 for the ‘All in’ compounds and 0.98 for the ‘Filled w/o Silane’ compounds. However, since each correlation is based on only three silica loadings, it is not statistically significant (*p* = 0.16 and *p* = 0.09, respectively) and should be regarded as an indication of a consistent trend. This trend is plausible, since both measurements are taken before significant crosslinking has occurred and therefore reflect a similar physical resistance to deformation. Mooney viscosity is therefore used here as an indicator of the compound viscosity contribution to cure torque, corresponding to *S*′*_min_* in the MDR curve. This is consistent with Lee et al. [9], who found the highest minimum torque for the silica-filled SSBR compound without silane and attributed it to filler clustering due to strong filler–filler interactions.

### 3.3. Crosslink Density

Following the approach outlined in Figure 2, the crosslink density of the cured rubber compounds was determined using equilibrium swelling analysis to isolate the contribution of chemical crosslinking to the overall cure torque.

Crosslink density measurements provide additional insight into the permanent network formed during vulcanization. However, in silica-filled systems, swelling behavior can still be affected by filler-related contributions, particularly strong filler–filler interactions, which may influence the apparent crosslink density determined from equilibrium swelling measurements, as reported by Bernal-Ortega et al. [30]. To account for the influence of filler content on the swelling behavior, the filler phr was subtracted from the total phr when calculating the crosslink density. However, even after this correction, the resulting crosslink density was still influenced by filler-related contributions (filler–filler and filler–polymer interactions), along with chemical crosslinking. As a result, the corrected crosslink density should still be regarded as an overestimation of the true chemical crosslink density, since physical filler network effects on swelling cannot be fully eliminated through this correction alone. Therefore, the reported values are referred to as apparent crosslink density, reflecting the combined contribution of chemical crosslinking and polymer–filler coupling reactions, in addition to the effect of the filler network that cannot be fully removed by the phr correction.

Table 5 presents the apparent crosslink density values for the unfilled compound, silica-filled compounds without silane (‘Filled w/o Silane’), and fully formulated compounds (‘All in’) at silica loadings of 30, 60, and 90 phr.

The unfilled compound exhibits the lowest crosslink density, as expected, since network formation arises only from sulfur crosslinking of the polymer chains without additional contributions from filler-related interactions. In the absence of silica, no filler–filler interactions are present that could influence the swelling behavior or restrict polymer mobility.

The compounds without silane exhibit crosslink density values that remain close to that of the unfilled compound across all silica loadings. Despite the substantially higher torque values observed in the MDR measurements and the increased compound stiffness reflected by the Mooney viscosity results, only minor changes in crosslink density are observed. Furthermore, a slight decrease in crosslink density is observed with increasing silica loading. This indicates that the increase in cure torque in these compounds does not originate from increased chemical crosslinking but rather from physical contributions, primarily strong filler–filler interactions and silica flocculation. The relatively constant crosslink density further indicates the absence of significant polymer–filler interactions in the compounds without silane. In these systems, the lack of a coupling agent prevents the formation of silica–polymer coupling, while the strong adsorption of accelerators on the silica surface can additionally reduce their availability for vulcanization. Consequently, although the compounds exhibit high torque values, particularly at high silica loadings, the extent of chemical crosslinking remains comparable to that of the unfilled compound. A similar observation was reported by Lee et al. [9] for an end-chain functionalized SSBR filled with 60 phr of the same silica grade (ULTRASIL^®^ 7000 GR). The compound without silane showed the lowest chemical crosslink density of all compounds, about 30% below that of the unfilled compound. The authors attributed this to adsorption of accelerators on the silica surface. Furthermore, this compound showed the largest torque increase during curing. This agrees with the present finding that a high cure torque in compounds without silane does not reflect a high chemical crosslink density.

The most pronounced increase in crosslink density is observed for the ‘All in’ compounds. In contrast to the compounds without silane, the crosslink density increases substantially with increasing silica loading, from 234 ± 12 mol/m^3^ at 30 phr to 342 ± 24 mol/m^3^ at 90 phr. This behavior indicates the contribution of silica–silane–polymer coupling reactions in addition to sulfur crosslinking. The increasing crosslink density with silica loading indicates that silane promotes coupling between the polymer and silica surface, restricting polymer mobility and reducing solvent uptake during swelling. Consequently, the apparent crosslink density increases with filler loading. Nevertheless, comparison with the MDR results shows that the increase in torque is significantly larger than the increase in crosslink density. For instance, the compounds without silane exhibit very high torque values despite maintaining crosslink densities close to the unfilled system. The increase in apparent crosslink density in the presence of silane is consistent with Lee et al. [9]. At 60 phr silica, they found that the apparent crosslink density increased in the order unfilled < TEOS (tetraethoxysilane) < without silane < TESPD < TESPT. Relative to the unfilled compound, their TESPD compound showed an apparent crosslink density about 2.1 times higher. This is similar to the ratio of 2.4 found here for the 60 phr ‘All in’ compound (274 vs. 114 mol/m^3^). Furthermore, the authors showed that the chemical crosslink density of the rubber matrix was almost identical for all silane-containing compounds. The increase in apparent crosslink density therefore originated from filler–rubber interactions, which accounted for 63% of the apparent crosslink density with TESPD and 70% with TESPT. This supports the interpretation in the present work that the increase in the apparent crosslink density of the ‘All in’ compounds with silica loading is mainly caused by silica–silane–polymer coupling rather than by additional sulfur crosslinks in the polymer matrix.

This comparison highlights an important aspect of interpreting cure behavior in silica-filled compounds: high cure torque does not necessarily indicate high chemical crosslink density. Instead, a substantial contribution may arise from both filler–polymer interactions (which contribute to the overall apparent crosslink density) and filler–filler interactions (which influence cure torque but are not reflected in the crosslink density), particularly at high silica loadings. Bernal-Ortega et al. [30] compared four methods for determining the crosslink density of silica-filled SBR compounds and showed that equilibrium swelling overestimates the crosslink density of filled compounds, since the non-swellable filler particles restrict the swelling of the rubber. For a compound with unmodified silica, they found a surprisingly high crosslink density, which they attributed to the strong filler–filler network, while compounds with TESPD showed considerably higher values due to the additional polymer–filler coupling. The same ranking is observed here. The compounds without silane show apparent crosslink densities above that of the unfilled compound, although accelerator adsorption would be expected to reduce the chemical crosslink density [9], whereas the ‘All in’ compounds show much higher values. This indicates that the non-silanized filler network contributes to the apparent crosslink density, but to a much smaller extent than silica–silane–polymer coupling.

### 3.4. Total Bound Rubber Content

Following the approach outlined in Figure 2, the bound rubber content (BRC) was used to evaluate the strength of filler–polymer interactions in the uncured compounds. Since BRC is measured on the uncured compound, it does not directly correspond to the cure torque recorded during MDR measurements, but it provides a useful indication of the filler–polymer interactions expected in the cured state. BRC was determined only for the ‘All in’ compounds: for the unfilled compound, the absence of filler makes BRC inapplicable, and for the compounds without silane, the separation of the bound rubber gel from the solvent could not be achieved. Table 6 presents the bound rubber results for the ‘All in’ compounds at increasing silica loadings.

BRC increases with increasing silica loading, indicating that the extent of filler–polymer interaction increases progressively with increasing filler content. Surprisingly, even at 30 phr, below the percolation threshold (~45 phr) required for a continuous filler network [13], a substantial bound rubber content is observed. This indicates that in the absence of a filler network, substantial polymer–filler interaction is present at this loading, promoted by both the silane and the end-chain functionalization of SPRINTAN^®^ 4602. The increase in bound rubber content with filler loading is consistent with Wolff et al. [19], who showed for different carbon black grades in SBR that the total bound rubber content increases with increasing filler loading and specific surface area. The absolute values are in the range reported for silica-filled SBR compounds containing a coupling agent. Choi and Ko [20] found total bound rubber contents of 26.0 to 63.5% for non-functionalized SSBRs with 50 phr silica and TESPT. Robertson et al. [8] reported about 67% for a non-functionalized SBR with 50 phr silica and a mercaptosilane coupling agent. In this work, a similar level (52%) is already reached at 30 phr, and 76% at 90 phr. The high value at low loading is attributed to the end-chain functionalization of SPRINTAN^®^ 4602. This is supported by Lin et al. [31], who found that SBRs with functional end groups gave substantially higher bound rubber contents (37.9% and 49.6%) than the corresponding non-functionalized polymer (27.5%). However, the total bound rubber content comprises both physically adsorbed and chemically bound rubber. After ammonia treatment, the chemically bound rubber accounted for the major part of the total bound rubber at 60 and 90 phr. At 30 phr, the compound did not remain intact during ammonia treatment, so no chemically bound rubber could be determined, which indicates that the bound rubber at this loading is mainly physically adsorbed. A clear separation of both contributions is nevertheless difficult in the uncured state. Silica–silane–polymer coupling takes place mainly during vulcanization, and rubber occluded within silica clusters may remain after ammonia treatment and thus be counted as chemically bound. The total bound rubber content is therefore used here as an indicator of the overall filler–polymer interaction rather than as a direct measure of chemical filler–polymer coupling.

Based on these BRC results, and considering the combined contributions to cure torque outlined in Figure 2, both filler–polymer and filler–filler interactions are expected to increasingly contribute to the higher cure torque observed at higher silica loadings.

### 3.5. Filler–Filler Interaction (G′ at Low Strain)

In silica-filled systems, the silanol groups on the silica surface promote re-clustering of filler over time, forming a filler network that increases compound stiffness and contributes to a further increase in torque during vulcanization [32]. The storage modulus at low strain, *G*′ (0.56 %), reflects the strength of this filler network and therefore provides a direct indication of the extent of filler–filler interactions in the compound. Because strong filler–filler interactions reduce the filler surface area available for silanization, filler flocculation competes with silanization and hinders the formation of effective filler–polymer interactions. Therefore, *G*′ (0.56%) helps to differentiate these filler-related interactions in the compounds. Table 7 presents the *G*′ (0.56%) results for the compounds without silane and the ‘All in’ compounds at increasing silica loadings.

At 30 phr, *G*′ is similar for the compounds without silane and the ‘All in’ compounds. Since this loading is below the percolation threshold (~45 phr) [13] required to form a continuous filler network, the filler remains as disconnected clusters instead of an interconnected network (irrespective of the presence of silane).

At 60 phr, however, a substantial difference is observed: *G*′ (0.56%) for the compound without silane is more than six times higher than for the corresponding ‘All in’ compound. The filler loading is above the percolation threshold, resulting in the presence of a continuous filler network. In the absence of silane, the silanol groups on the silica surface remain freely available to interact with each other via hydrogen bonding, allowing the formation of an extensive filler–filler network, leading to a substantial increase in *G*′. In the ‘All in’ compound, silane acts as a bridge between the silica surface and the polymer, reacting with the silanol groups during mixing (silanization reaction). This reduces the number of silanol groups available for filler–filler interactions and instead promotes filler–polymer coupling reactions. As a result, the filler network formation in the ‘All in’ compound is suppressed, indicated by significantly lower *G*′ (0.56%) value.

At 90 phr, these differences become even more pronounced: *G*′ (0.56%) for the compound without silane is ten times higher than for the corresponding ‘All in’ compound. With increasing silica loading, more silanol groups become available on the additional filler surface, providing a greater extent of filler–filler interactions in the absence of silane. In the ‘All in’ compound, silane continues to react with the additional silanol groups at the increased filler loading, again promoting filler–polymer coupling reactions over filler–filler interactions. As a result, filler network formation continues to be suppressed at this loading, indicated by a significantly lower *G*′ (0.56%) value.

These results show that silane increasingly suppresses filler–filler interaction as silica loading increases. Furthermore, in the absence of silane, the lack of such suppression results in strong filler–filler interactions, indicated by high *G*′ (0.56%) values. This indicates that at higher silica loadings, the increase in cure torque in the absence of silane reflects a strong filler network and not necessarily an increase in chemical crosslinking. These results are consistent with the to the literature. The strong increase in *G*′ (0.56%) without silane, mainly between 30 and 60 phr, is consistent with Luginsland et al. [28], who found a significant Payne effect in silica compounds without silane from loadings of 60 phr and above. The significantly lower *G*′ (0.56%) of the ‘All in’ compounds is consistent with Robertson et al. [8] and Lin et al. [7], who showed that silanes strongly suppress silica flocculation.

## 4. Filler-Dependent Subdivision of Cure Torque

To gain a deeper understanding of the mechanisms contributing to torque development during vulcanization, a comprehensive subdivision of the MDR cure torque was performed for the ‘All in’ compounds filled with varying silica contents. The goal of this subdivision is to estimate and compare the contributions of four key mechanisms that influence the torque response during curing:

- Compound viscosity (related to the uncured flow resistance).
- Chemical crosslinking (covalent network formation).
- Filler–filler interactions (physical flocculation and silica networking).
- Filler–polymer coupling (chemical bridging facilitated by silane between filler and polymer chains).

Table 8 lists the measured values and serves as the input dataset for the subdivision framework. The table enables a clear comparison across silica loadings and provides a consistent basis for assigning torque contributions to the different mechanisms.

**Figure 5:**
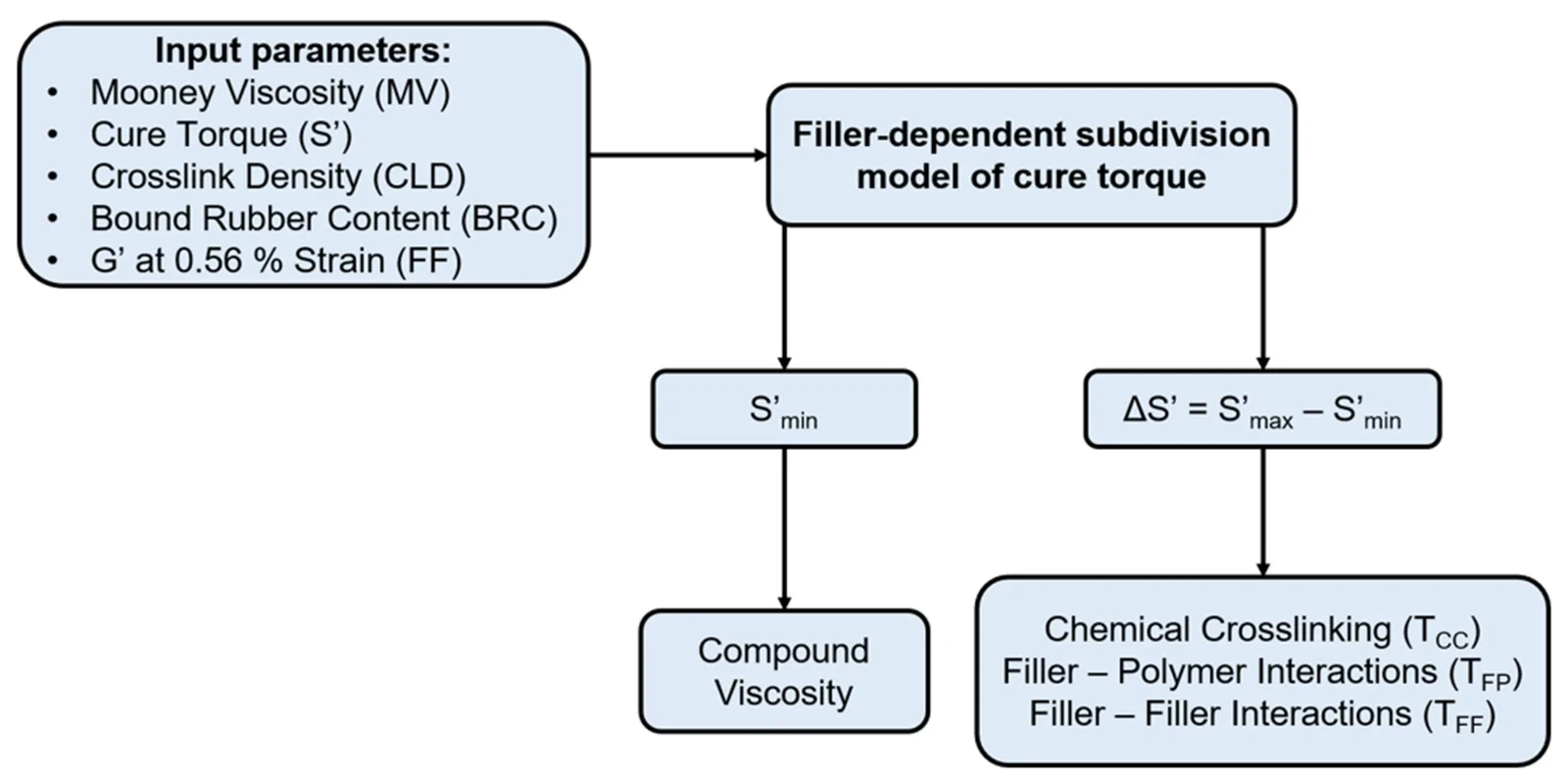
Filler-dependent subdivision model for MDR torque analysis.

Torque Subdivision for 90 phr silica-filled ‘All in’ compound:

To present the subdivision procedure, the 90 phr filled ‘All in’ compound is used as an example. The compound viscosity, as shown in Section 3.2, is related to the *S*′*_min_* from the cure torque. Furthermore, the total torque increase during vulcanization (Δ*S*′ = *S*′*_max_* – *S*′*_min_*) is attributed to the combined effects of chemical crosslinking (CC) and filler-related contributions (filler–polymer (FP) and filler–filler (FF) interactions). The subdivision is then performed using a normalized share-based approach derived from independently measured parameters listed in Table 8. Figure 5 presents a flowchart of the torque subdivision framework, indicating how the overall torque response is separated into distinct contributions based on experimentally measured input parameters.

The subdivision is based on the concept that the modulus of a filled rubber compound is the sum of contributions from the polymer network, the hydrodynamic effect, filler–polymer interactions and filler–filler interactions [28,29]. Accordingly, the torque rise Δ*S*′ is treated as the sum of the contributions of chemical crosslinking, filler–polymer interactions and filler–filler interactions, represented by CLD, BRC and *G*′ (0.56%), respectively. Since these indicators have different units, each is normalized to its maximum within the series, providing dimensionless values that describe the relative change in each mechanism with silica loading. This assumes that each contribution is proportional to its indicator and that the three normalized indicators have the same sensitivity to torque. As the sensitivity cannot be verified independently, the absolute torque values are estimates.

The torque subdivision is carried out in the following steps:

**Step 1:** Compound Viscosity (Baseline Torque Level)

The torque prior to the onset of curing is represented by the minimum torque value **S*′*_min_**, which is aligned with the Mooney viscosity ML (1 + 4) 100 °C. This value reflects the compound’s flow resistance and acts as the reference point against which the torque increase from network formation and other interactions during curing is measured. For the 90 phr ‘All in’ compound, this value is given by Equation (6):

(6) **S*′*_min_** = 3.1 dNm

**Step 2:** Total Torque Rise

For the 90 phr filled ‘All in’ compound, the total torque rise is calculated using Equation (7):

(7) $${\Delta S^{\prime }=\;{S^{\prime }}_{\max}-\;S^{\prime }}_{\min}=18.6\;\mathrm{dNm}-3.1\;\mathrm{dNm}=15.5\;\mathrm{dNm}\;$$

**Step 3:** Normalize the Contributing Parameters

For this compound, the following experimentally measured quantities were used to determine the relative share of each torque contribution (CC, FP and FF):

- Apparent crosslink density (CLD) = 342 mol/m^3^.
- Bound rubber content (BRC) = 76%.
- Storage modulus at low strain, *G*′ (0.56%) = 1200 kPa.

These values are normalized against the maximum across all systems and are given by Equations (8)–(10):

(8) $$\mathrm{Normalized}\;\mathrm{weight}\;\mathrm{due}\;\mathrm{to}\;\mathrm{CLD}\;=\;w_{\mathrm{CLD}}=\frac{342}{\max\;(\mathrm{CLD})}=\;\frac{342}{342}=1.000$$

(9) $$\mathrm{Normalized}\;\mathrm{weight}\;\mathrm{due}\;\mathrm{to}\;\mathrm{BRC}\;=\;w_{\mathrm{BRC}}=\frac{76}{\max\;(\mathrm{BRC})}=\frac{76}{76}=1.000$$

(10) $$\mathrm{Normalized}\;\mathrm{weight}\;\mathrm{due}\;\mathrm{to}\;G^{\prime }\;=w_{G^{\prime }}=\;\frac{1200}{\max\;(G^{\prime })}=\frac{1200}{1200}=1.000$$

Therefore, the total normalized weight (*W_total_*) is given by Equation (11):

(11) *W_total_* = *w_CLD_* + *w_BRC_* + *w_*G*′_* = 1.000 + 1.000 + 1.000 = 3.000

**Step 4:** Allocate Torque Proportionally

Each component’s torque share was calculated based on its normalized weight fraction, as indicated by Equations (12)–(14):

(12) $$\mathrm{Chemical}\;\mathrm{Crosslinking}\;\mathrm{Torque}\;=\;T_{\mathrm{CC}}=\;\Delta S^{\prime }\;\times \;\frac{w_{\mathrm{CLD}}}{W_{\mathrm{total}}}=15.5\;\times \;\frac{1.000}{3.000}\;=\;5.17\;\mathrm{dNm}$$

(13) $$\mathrm{Filler}\text{–}\mathrm{Polymer}\;\mathrm{Interaction}\;\mathrm{Torque}\;=\;T_{\mathrm{FP}}\;=\Delta S^{\prime }\;\times \;\frac{w_{\mathrm{BRC}}}{W_{\mathrm{total}}}=15.5\;\times \;\frac{1.000}{3.000}\;=\;5.17\;\mathrm{dNm}$$

(14) $$\mathrm{Filler}\text{–}\mathrm{Filler}\;\mathrm{Interaction}\;\mathrm{Torque}\;=\;T_{\mathrm{FF}}\;=\Delta S^{\prime }\;\times \;\frac{w_{G^{\prime }}}{W_{\mathrm{total}}}=15.5\;\times \;\frac{1.000}{3.000}\;=\;5.17\;\mathrm{dNm}$$

**Step 5:** Consistency Check

The final torque value (*S*′*_max_*) can be recalculated by adding the minimum torque (*S*′*_min_*) and the torque contributions from the three individual mechanisms: chemical crosslinking, filler–polymer interactions, and filler–filler interactions. This is given by Equation (15):

(15) $${{S^{\prime }}_{\max}=S^{\prime }}_{\min}+T_{\mathrm{CC}}+T_{\mathrm{FP}}+T_{\mathrm{FF}}=\left(3.10+\;5.17+5.17+5.17\right)\mathrm{dNm}=18.6\;\mathrm{dNm}$$

**Figure 6:**
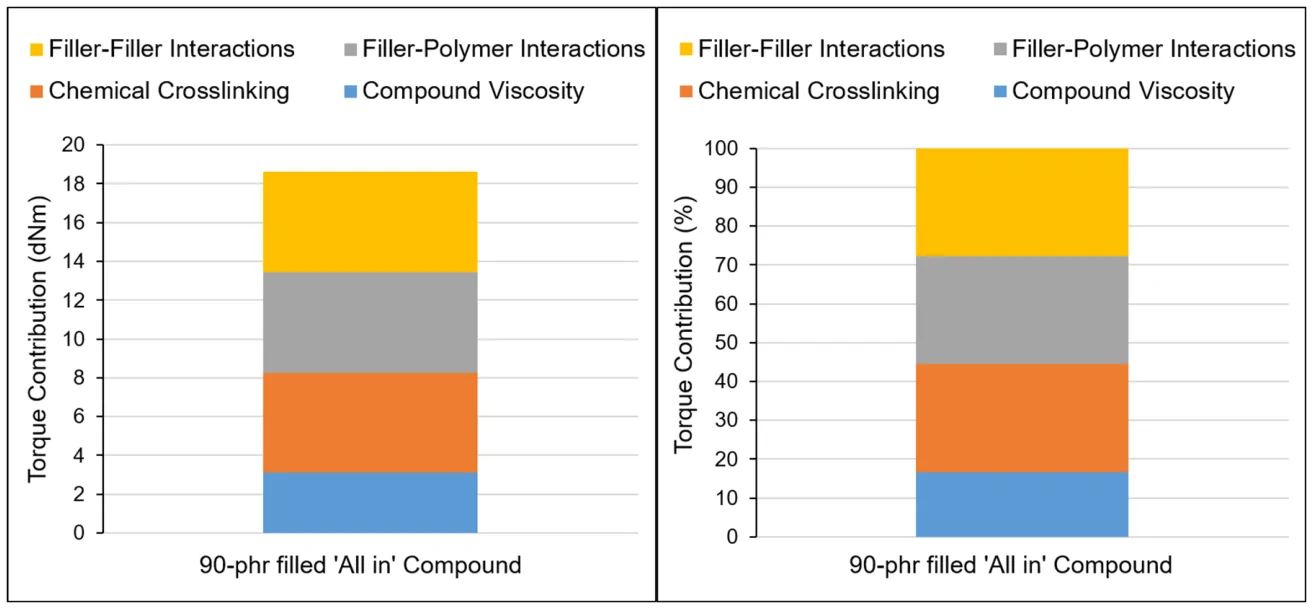
Torque subdivision for ‘All in’ compound filled with 90 phr silica: (**left**) absolute torque values; (**right**) normalized torque values.

This matches the experimentally measured maximum torque for the 90 phr ‘All in’ compound. However, this result is expected, since the three torque contributions were obtained by dividing Δ*S*′ into different parts, and hence their sum is equal to Δ*S*′. It therefore confirms the consistency of the calculation, but does not provide an independent validation of the model. Figure 6 presents the subdivision of torque contributions for this compound. It illustrates the breakdown of the final cure torque into four primary components: compound viscosity (*S*′*_min_*), chemical crosslinking, filler–polymer coupling, and filler–filler interactions. This visual representation reflects both the absolute magnitude of each component and its normalized proportions within the total torque.

At 90 phr, the three mechanisms (chemical crosslinking, filler–polymer coupling, and filler–filler interactions) are each assigned approximately one-third of Δ*S*′. This is a direct consequence of the normalization approach used: since 90 phr represents the highest silica loading investigated, it also corresponds to the highest measured value for CLD, BRC, and *G*′ (0.56%) within this dataset, so each parameter reaches its own maximum simultaneously at this loading. While this equal split does not indicate which mechanism is dominant, it reflects that all three contributions have increased substantially in the highest loading investigated, consistent with the sharp increases in CLD, BRC, and *G*′ (0.56%) values observed between 60 and 90 phr.

**Figure 7:**
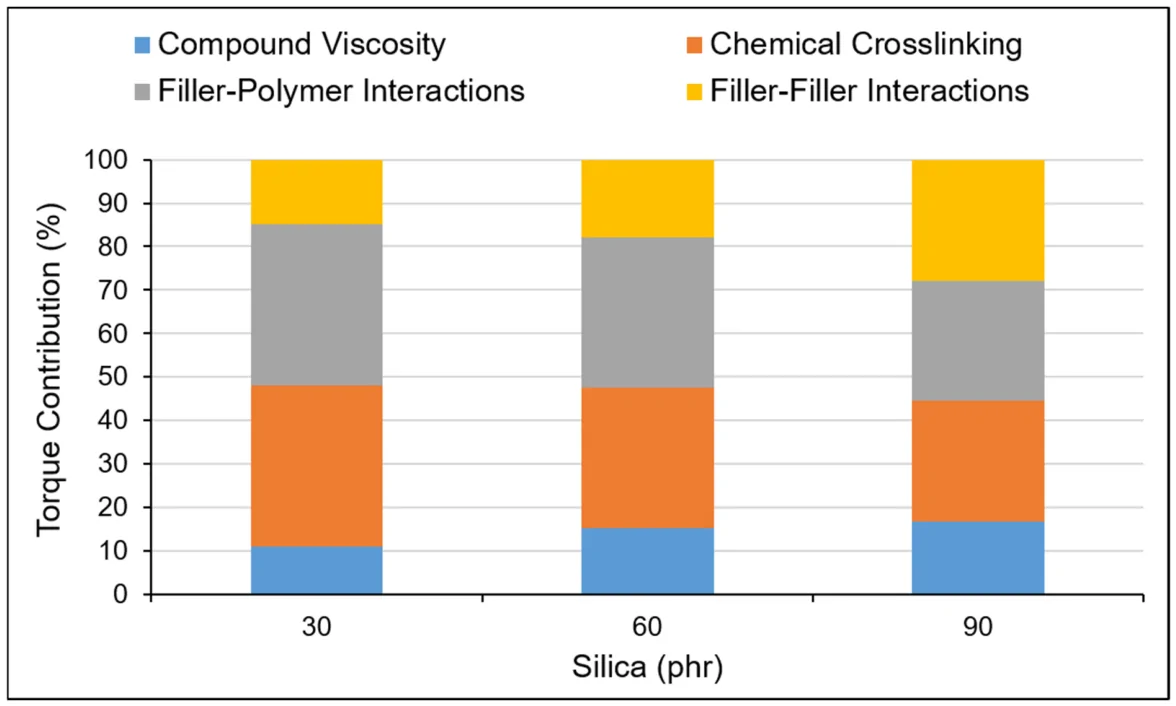
Normalized torque subdivision for ‘All in’ compounds at 30, 60, and 90 phr silica loading.

Building on the subdivision done for the 90 phr filled SPRINTAN^®^ 4602 compound, the same methodology was applied to the 30 and 60 phr filled ‘All in’ compounds. This enables a comparison of how torque contributions change with increasing silica loading. To maintain comparability across filler loadings, the analysis is based on normalized torque values. Figure 7 presents the normalized torque subdivision for ‘All in’ compounds across the three silica loadings.

The relative contributions of compound viscosity, chemical crosslinking, filler–polymer interactions, and filler–filler interactions shift with increasing filler loading. At 30 phr, chemical crosslinking and filler–polymer interactions dominate in near-equal proportions (37.1% and 37.0% of *S*′*_max_*, respectively), consistent with the measurable bound rubber content observed at this loading. The contribution of filler–filler interactions is small (14.9%), indicating the presence of limited and isolated clusters of filler network below the percolation threshold (~45 phr).

At 60 phr, the distribution becomes more balanced: filler–polymer interactions account for 34.7%, chemical crosslinking remains substantial at 32.5%, and the filler–filler contribution increases to 17.8%. This indicates continued bound rubber formation alongside the onset of a more developed filler network above the percolation threshold (~45 phr), consistent with the rise in *G*′ (0.56%) between 30 and 60 phr. At 90 phr, all three mechanisms exhibit an approximately equal weightage (~28% each). This is a direct consequence of 90 phr representing the highest silica loading investigated: CLD, BRC, and *G*′ (0.56%) each reach their maximum value at this loading. The normalization approach assigns them comparable weight and does not differentiate the dominance of one mechanism over the others at this loading.

In summary, the torque subdivision analysis across the three silica loadings of the ‘All in’ compounds shows a clear trend. At low filler loading, chemical crosslinking and filler–polymer interactions dominate the cure torque. As silica loading increases, filler-related contributions become increasingly significant, whereas the equal shares obtained at the highest loading result from the normalization and do not allow the dominant mechanism to be identified. Though torque rise (Δ*S*′) is often attributed primarily to chemical crosslinking, the results show that its relative contribution to total torque decreases with increasing filler content, while the combined contribution from filler-related interactions increases correspondingly. This confirms that torque rise cannot be directly correlated with crosslink density in silica-filled systems, a misconception often made in traditional rubber analysis.

While the normalized subdivision model provides a useful framework for isolating and comparing these contributions, it remains a simplification of a highly complex system. The torque contributions are estimated based on relative scaling of experimentally measured parameters (CLD, BRC, and *G*′ (0.56%)) and may not fully capture the actual or overlapping effects, particularly at high filler loadings where the interactions may become more complex and influence one another. Nevertheless, the method provides a semi-quantitative estimate of the different mechanisms involved in torque development and shows how their relative contribution changes with filler content.

## 5. Conclusions

This paper aimed to subdivide the maximum cure torque of silica-filled SSBR compounds into its individual contributions, in order to clarify the extent to which the commonly assumed torque–crosslink density correlation holds in such systems. The curing behavior of the unfilled compound, the silica-filled compounds without silane, and the ‘All in’ compounds based on SPRINTAN^®^ 4602 was investigated and compared across increasing silica loadings. A filler-dependent subdivision model that estimated the relative contributions of compound viscosity, chemical crosslinking, filler–polymer interactions, and filler–filler interactions to the cure torque was presented.

In the absence of silane, increasing silica loading resulted in a significant increase in viscosity and cure torque, particularly at high filler loadings. However, the corresponding crosslink density remained close to that of the unfilled compound and even showed a slight decrease with increasing silica content. This indicates that the increased torque response does not reflect increasing chemical crosslinking but strong filler–filler interactions and filler flocculation. Therefore, the high torque values observed for the compounds without silane can be associated with physical contributions instead of the formation of additional covalent crosslinks. The incorporation of silane significantly changed the curing behavior. The ‘All in’ compounds exhibited lower viscosities and reduced torque values compared to the corresponding compounds without silane, indicating suppression of filler–filler interactions and shielding of filler by silane. At the same time, the apparent crosslink density increased progressively with increasing silica loading, indicating the contribution of filler–polymer interactions in addition to sulfur crosslinking. Furthermore, even in the presence of silane, filler-related contributions remained significant at high silica loadings, particularly at 90 phr.

The combined interpretation of viscosity, cure behavior, apparent crosslink density, and the storage modulus at low strain, together with the torque subdivision model, demonstrates that cure torque is governed primarily by chemical crosslinking at low filler loadings and increasingly by filler-related contributions at high filler loadings. Consequently, the measured cure torque cannot be directly interpreted as a measure of chemical crosslink density, particularly in highly filled silica compounds where physical interactions contribute substantially to the torque response. These results were obtained for the investigated functionalized SSBR. It is expected that this outcome can be transferred to other systems. However, this needs to be verified in future studies. These findings provide qualitative and semi-quantitative insight into the mechanisms governing cure torque development in silica-filled SSBR compounds and contribute to improved interpretation of MDR results for formulation design and optimization of silica-filled rubber compounds for demanding applications such as tire tread systems.

## Figures and Tables

**Table 1:** List of ingredients.

Ingredients		Role
SPRINTAN^®^ 4602	SSBR	Polymer
ULTRASIL^®^ 7000 GR	Silica	Filler
Si 266^®^	Silane	Silane coupling agent
Edenor ST1 GS	Stearic Acid	Activators
ZnO *RS RAL 844 C*	Zinc Oxide	
Vulkanox 4020/LG	6PPD	Antioxidant
Protektor G 3108	Wax	Antiozonant
N330	Carbon Black	Antioxidant and colorant
Vivatec 500	TDAE Oil	Lubricant
α-Sulphur	Sulfur	Vulcanization system
Rhenogran TBBS-80	TBBS	
Rhenogran DPG	DPG	
Richon TBZTD OP	TBzTD	

**Table 2:** Compound formulation (phr).

Ingredients	Unfilled	Filled Without Silane	All in
SSBR	100	100	100
Silica	-	30/60/90	30/60/90
Silane	-	-	2.2/4.4/6.5
Zinc Oxide, Stearic Acid, 6PPD, Wax	2 each	2 each	2 each
Carbon Black	3	3	3
TDAE Oil	-	9.5/19/28	9.5/19/28
Sulfur	1.4	1.4	1.4
TBBS, DPG	1.5 each	1.5 each	1.5 each
TBzTD	0.4	0.4	0.4

**Table 3:** Mixing procedure.

**1st Stage**		
Fill Factor: 72% Initial temperature: 80 °C Initial rotor speed: 70 rpm	Time (mm:ss)	Workflow
	00:00	Add raw rubber; start measurement
	00:20	Ram down
	01:00	Ram up, add 2/3 (silica + silane)
	01:30	Ram down
	02:30	Ram up, add oil, chemicals, 1/3 (silica + silane)
	03:00	Ram down
	04:00	Ram up, sweep for 15 s
	04:15	Ram down, isothermal mixing at 140 °C
	07:00	Dump and check weight, sheet out on mill × 5
**2nd Stage**		
Fill Factor: 69% Initial temperature: 80 °C Initial rotor speed: 80 rpm	00:00	Add batch stage 1, ram down; start measurement
	00:50	Ram up, add DPG
	01:00	Ram down, adjust speed, isothermal mixing at 140 °C
	05:00	Dump and check weight, dump T, sheet out on mill × 5
**3rd Stage**		
Fill Factor: 66% Initial temperature: 50 °C Initial rotor speed: 50 rpm	00:00	Add batch stage 2, ram down; start measurement
	00:30	Ram up, add vulcanization system, ram down
	03:00	Dump and check weight, sheet out on mill × 5

**Table 4:** Mooney viscosity results.

Mooney Viscosity ML (1 + 4) 100 °C (MU)			
Silica (phr)	Unfilled	Filled w/o Silane	All in
0	48	-	-
30	-	78	73
60	-	123	88
90	-	179	92

**Table 5:** Crosslink density results.

Crosslink Density (mol/m^3^)			
Silica (phr)	Unfilled	Filled w/o Silane	All in
0	114 ± 3	-	-
30	-	153 ± 11	234 ± 12
60	-	142 ± 9	274 ± 19
90	-	141 ± 8	342 ± 24

**Table 6:** Total bound rubber content results.

Total Bound Rubber Content (%)	
Silica (phr)	All in
0	-
30	52 ± 0.1
60	65 ± 0.2
90	76 ± 0.6

**Table 7:** Results for storage modulus at low strain, *G*′ (0.56%).

	Filled w/o Silane	All in
Silica (phr)	*G*′ (kPa) at 0.56%	
0	**-**	**-**
30	459	332
60	3347	527
90	12,228	1200

**Table 8:** Experimental parameters used for torque subdivision.

Filler (phr)	Compound Viscosity (MU)	*S*′*_min_* (dNm)	*S*′*_max_* (dNm)	CLD (mol/m^3^)	Total BRC (%)	*G*′ (0.56%) (kPa)
30	73	1.5	13.6	234.7	52	332
60	88	2.4	15.9	274.2	65	527
90	92	3.1	18.6	342.0	76	1200

## Data Availability

The raw data supporting the conclusions of this article will be made available by the authors on request.
